# Laparoscopic repair of secondary parahiatal hernia with incarceration of the stomach: a case report

**DOI:** 10.1186/1752-1947-7-50

**Published:** 2013-02-19

**Authors:** Masashi Takemura, Katsuyuki Mayumi, Takashi Ikebe, Genya Hamano

**Affiliations:** 1 Department of Surgery, Gohshi Hospital, 1-8-20, Nagasu Nishi-Dori, Amagasaki, Hyogo 660-0807, Japan; 2 Department of Upper Gastrointestinal Surgery, Hyogo College of Medicine, 1-1, Mucogawa-machi, Nishinomiya, Hyogo 663-8501, Japan

## Abstract

**Introduction:**

Parahiatal hernia is an extremely rare subtype of hiatal hernia, which in turn is a type of diaphragmatic hernia in adults, and only a few cases have been reported to date. We report the case of a patient who suffered from gastric incarceration through an anatomically separate diaphragmatic defect, immediately lateral to a structurally normal esophageal hiatus, that developed after treatment of a malignant mesothelioma.

**Case presentation:**

A 70-year-old Japanese man, who had undergone treatment for a left malignant pleural mesothelioma a year ago at another hospital, was referred to our institution following a 4-day history of epigastric pain. Esophagogastroscopy demonstrated a normal esophagogastric junction, with remarkable stenosis and active gastric ulcer of the gastric body. Histopathological examination of the gastric biopsy specimen confirmed a gastric ulcer. Furthermore, computed tomography revealed a large fluid-filled structure in the retrocardiac space. On the basis of preoperative data, we decided to attempt laparoscopic repair for the gastric volvulus. During surgery, gastric and omental herniation was observed within a peritoneal lined defect immediately lateral to the esophageal hiatus. Dissection near the esophageal hiatus revealed a discrete extrahiatal defect 3cm in diameter immediately adjacent to the left crus of the diaphragm. The parahiatal defect was closed using interrupted nonabsorbable heavy suture. The patient’s postoperative course was uneventful, and anastomotic leakage was not observed at postoperative barium swallowing.

**Conclusions:**

Although preoperative diagnosis of parahiatal hernia is difficult, a laparoscopic approach can be a useful therapeutic procedure not only for paraesophageal hernia but also for parahiatal hernia.

## Introduction

Parahiatal hernia is an extremely rare subtype of hiatal hernia, which in turn is a type of diaphragmatic hernia in adults. Parahiatal hernia is characterized by the presence of a diaphragmatic hernia defect immediately adjacent to an anatomically normal esophageal hiatus. Few cases of laparoscopic repair for parahiatal hernia have been reported to date [1]. Here we describe the case of a 70-year-old man who suffered from gastric incarceration through an anatomically separate diaphragmatic defect lateral to a structurally normal esophageal hiatus and discuss the laparoscopic management of such defects.

## Case presentation

A 70-year-old Japanese man was referred to our institution following a 4-day history of epigastric pain. He also complained of acute dysphagia and frequent episodes of severe vomiting. He had been diagnosed with a left malignant pleural mesothelioma and for the past year he had been treated at another hospital. At that time, a chest roentgenogram obtained before treatment for malignant mesothelioma revealed no elevation of the diaphragm. At the current admission, his body mass index was 18.3, and he appeared slightly emaciated. Decreased breath sounds and dullness on percussion of the left lower hemithorax were noted. His white blood cell count was 8100/uL, and his C-reactive protein level was 3.16mg/dL.

**Figure 1:**
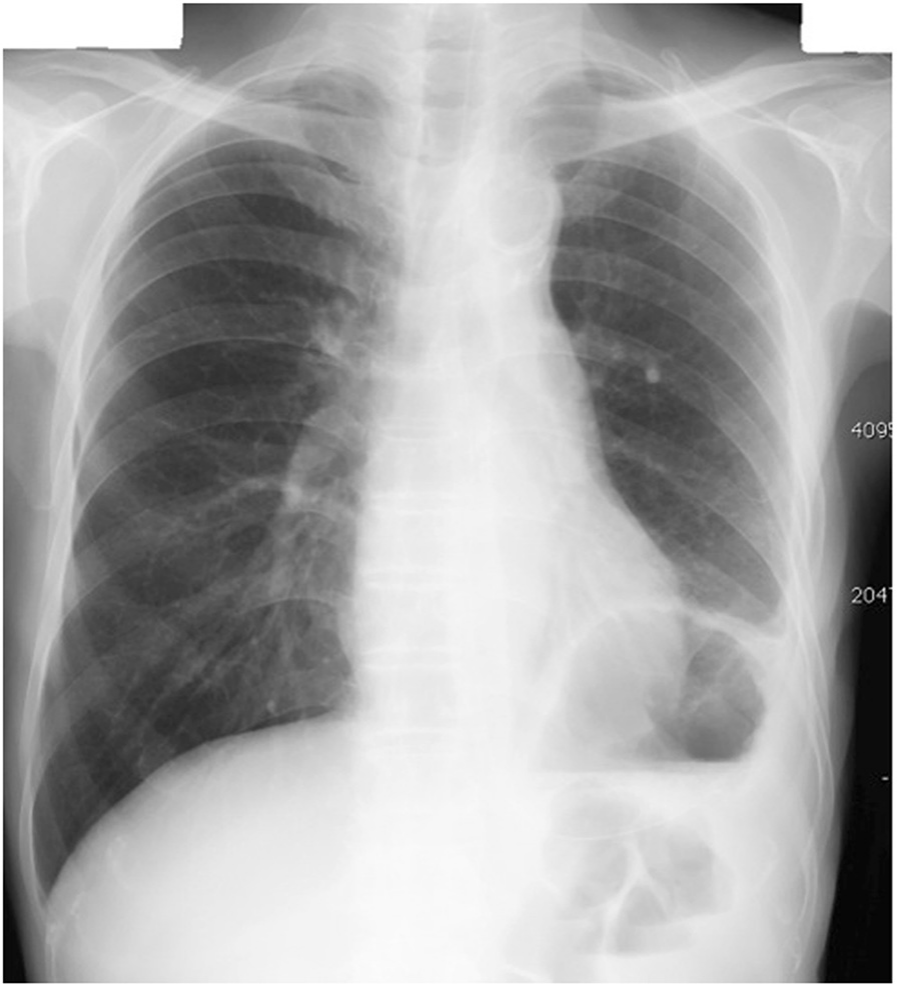
A preoperative chest roentgenogram demonstrating intrathoracic air–fluid level in the left chest.

**Figure 2:**
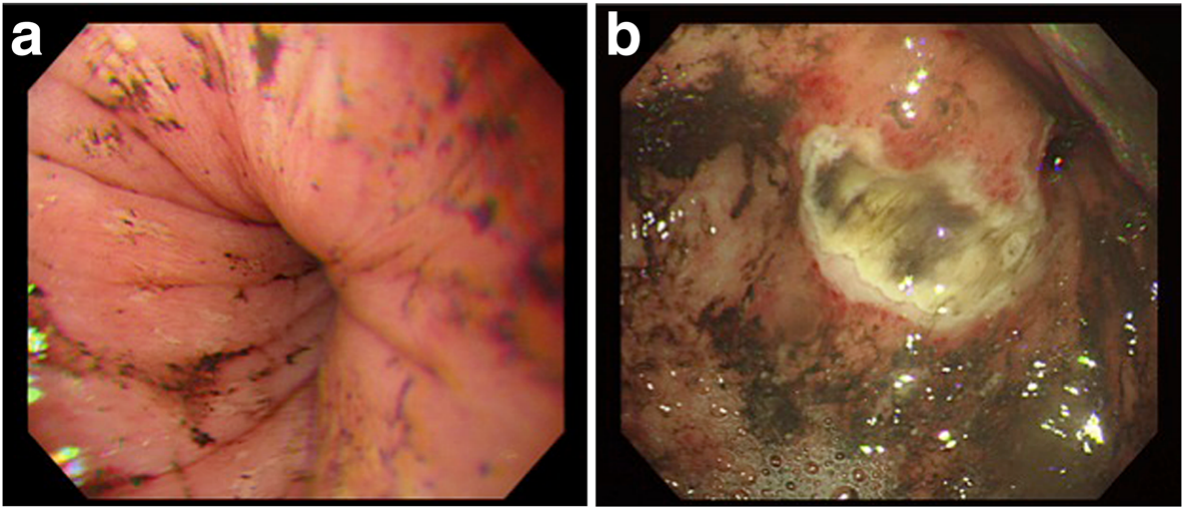
**Upper esophagogastroscopy demonstrating remarkable stenosis of gastric body (a) and an active ulcerative lesion in the middle gastric body (b). **Herniation of the gastric cardia and fundus are not detected.

**Figure 3:**
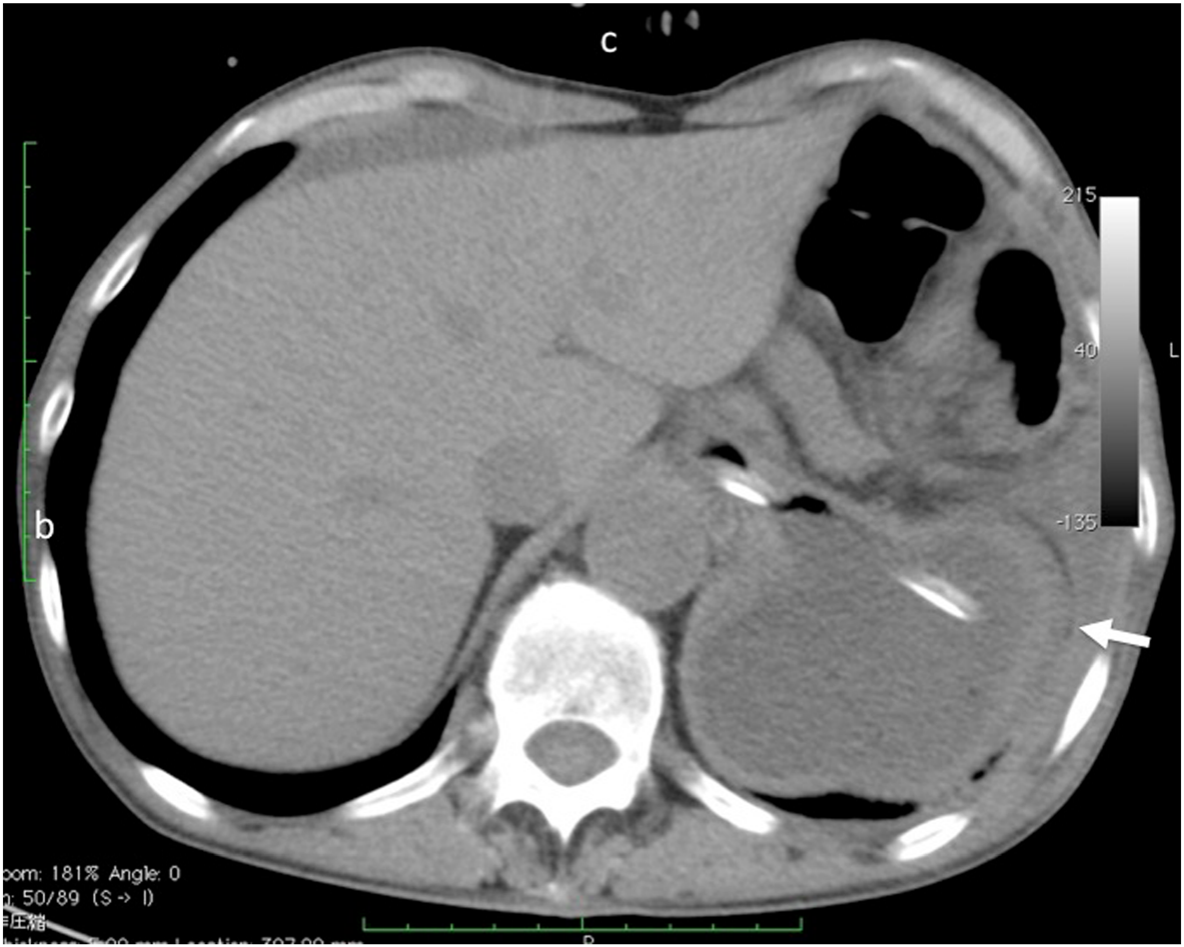
**Chest computed tomography showing a large fluid-filled structure in the retrocardiac space. **Mild left pleural effusion and atelectasis are indicated.

A chest roentgenogram revealed elevation of the left hemidiaphragm (Figure 1). Esophagogastroscopy demonstrated a normal esophagogastric junction, and remarkable stenosis of the gastric body. Although a hiatal hernia was not detected, an active ulcerative lesion was observed at the middle gastric body (Figure 2). Histopathological examination of a gastric biopsy specimen confirmed a gastric ulcer. Furthermore, computed tomography revealed a large fluid-filled structure in the retrocardiac space (Figure 3). Mild left pleural effusion and atelectasis were also detected. Ascites was not observed in the abdominal cavity. On the basis of these preoperative data, we decided to attempt laparoscopic repair for gastric volvulus.

**Figure 4:**
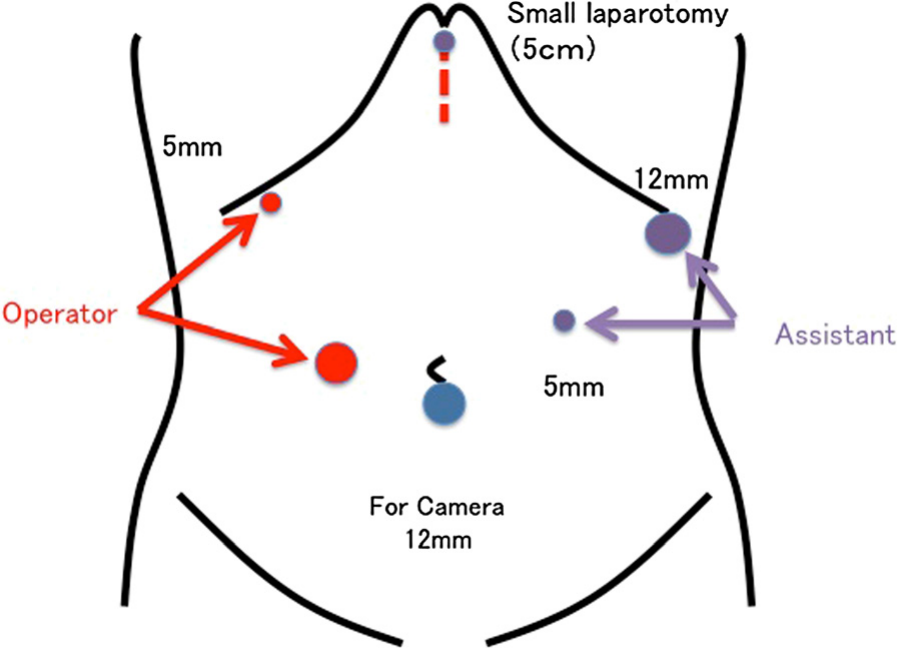
**The port positions used in our standard approach for laparoscopic distal gastrectomy. **A total of six ports were placed in the abdomen.

After general anesthesia induction and carbon dioxide insufflation of the abdominal cavity, laparoscopic exploration was performed. The standard approach for laparoscopic distal gastrectomy employed at our institution was used for this patient. A total of six ports were placed in the abdomen (Figure 4), and no additional ports were necessary.

**Figure 5:**
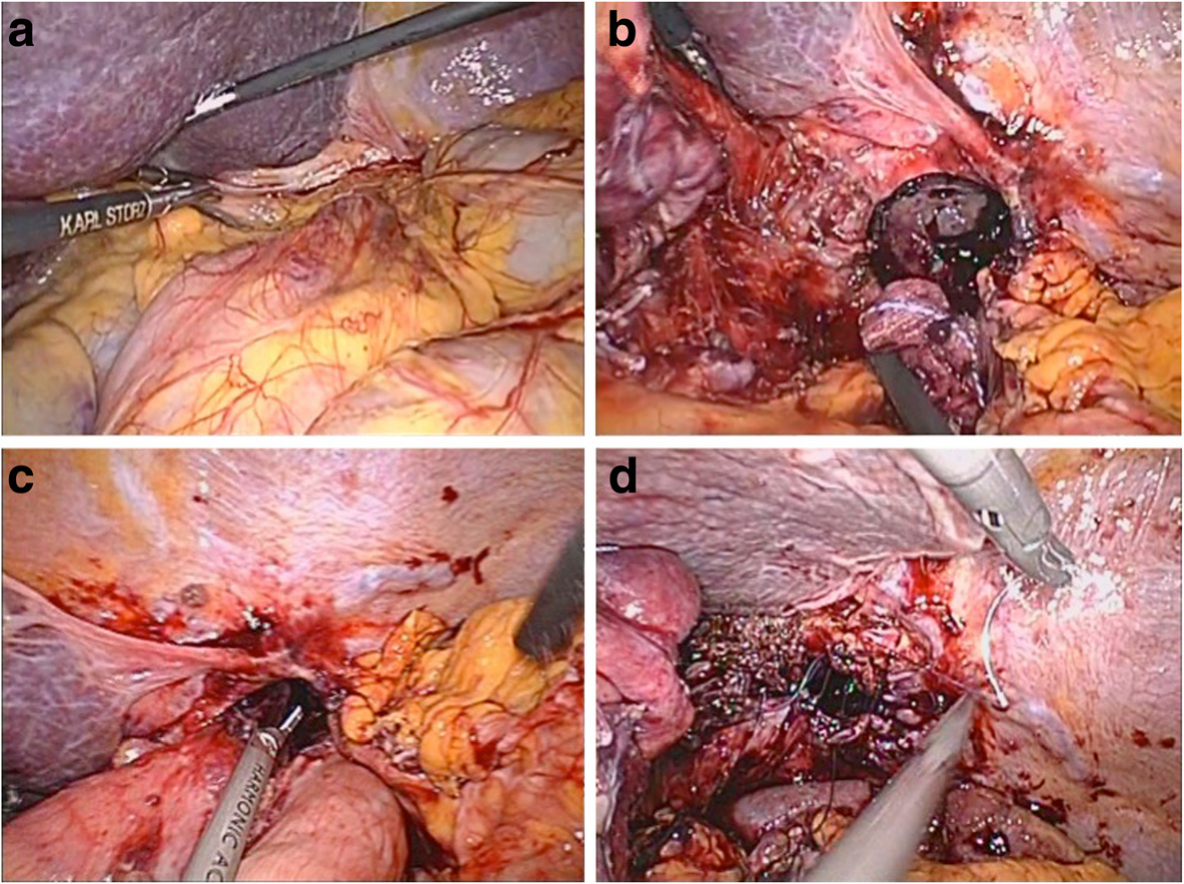
**The operative findings of our case. **Gastric and omental herniation is observed within a peritoneal-lined defect immediately lateral to the esophageal hiatus (**a**). The defect is clearly separated from the esophageal hiatus by the left crus of the diaphragm (**b**). The top of the gastric fundus, which was incarcerated, is friable (**c**). Interrupted nonabsorbable heavy sutures are used for closure of the parahiatal defect (**d**).

During surgery, gastric and omental herniation through a peritoneal-lined defect immediately lateral to the esophageal hiatus was observed (Figure 5a). This parahiatal defect was clearly separated from the esophageal hiatus by the muscular substance of the left crus of the diaphragm (Figure 5b). The stomach was grasped with laparoscopic forceps and repositioned into the abdomen. The top of the gastric fundus, which was incarcerated, was friable (Figure 5c). There was no evidence of necrosis of the stomach. During manipulation, a large full-thickness tear occurred in the gastric fornix.

Dissection near the esophageal hiatus revealed a discrete 3-cm diameter, extrahiatal defect immediately adjacent to the left crus of the diaphragm. The hernia sac was present within this defect, which had invaginated into the left retrocardiac space. Closure of the parahiatal defect was performed using monofilament absorbable suture (0 PDS™ [polydioxanone] II) (Figure 5d). Simultaneously, proximal gastrectomy was performed for the large tear in the fornix. Double tract reconstruction using a pedicled jejunum (esophagojejunostomy and jejunogastrostomy) was performed via an antecolic route. A laparoscopic antireflux surgery was not added. The total surgical duration was 215 minutes, and the amount of blood loss was minimal.

The patient’s postoperative course was uneventful, and anastomotic leakage was not observed during a postoperative barium swallowing. The patient resumed oral intake of food 5 days after surgery, and he was discharged from our hospital 29 days after surgery.

## Discussion

The most generally accepted nomenclature of hiatus hernia includes four categories: Type I (sliding hernia) which accounts for approximately 95% of cases of hiatal hernia, Type II (paraesophageal hernia) which accounts for approximately 5% of cases of hiatal hernias, and Type III which is a combination of both Type I and II. Type IV, parahiatal hernia, is also gaining widespread acceptance in the literature. Parahiatal hernia (Type IV) is differentiated from the foregoing three types (Types I to III) of hernia by the presence of a separate extrahiatal diaphragmatic defect with intervening normal crural muscle [2,3]. However, parahiatal hernia is extremely rare; the exact incidence of parahiatal hernia is not known. Only a few patients with this condition have been reported so far [1,2,4-6]. Palanivelu *et al*. [1] reported that the incidence of parahiatal hernia in their study on fundoplication for paraesophageal hernia was 0.35%.

Parahiatal hernia is characterized by the presence of a separate extrahiatal diaphragmatic defect with intervening normal crural muscle. Moreover, the hiatus is structurally normal and both crural muscles are intact [7,8]. In our patient, the esophageal hiatus was normal, and the hernia defect was separated from the hiatus by the left crus of the diaphragm at laparoscopic findings. These findings supported the diagnosis of parahiatal hernia in our patient.

Incomplete obliteration of the embryonic pleuroperitoneal canal, resulting in a persistent pneumoenteric recess, has been theorized to explain the etiology of naturally occurring primary parahiatal hernias [9]. Although these hernias may arise from both sides of the pneumoenteric recesses, they are usually found on the left side. This may be attributed to the presence of the liver on the right side, leading to protection of the diaphragm on that side [2,4-6]. However, the low incidence of this condition makes it difficult to draw firm conclusions. Secondary or acquired parahiatal hernia occurs as a result of protrusion of the gastric fundus through an intracrural defect, probably caused by disruption during crural repair for gastroesophageal reflux disease [1]. Moreover, secondary parahiatal hernia is also known to occur after esophagectomy, probably because of excessive manipulation of the crural muscles [10]. The secondary type is probably more common than the primary or congenital type. In our case, the left hemidiaphragm was not elevated before treatment of the left malignant pleural mesothelioma, whereas an elevation was observed after treatment. From these facts, we hypothesized that the mesothelioma developed at a fragile site of the left diaphragm, such as the left pneumoenteric recess, which then may have caused the hernial orifice in our patient. Papavramidis *et al*. described that chronically increased intra-abdominal pressure (IAP) leads to both morphological and biochemical adaptations of the costal diaphragm [11]. Chronic IAP, such as obesity and ascites and so on, was reported to play an important role in the causes of a hernia at the weak point of the abdominal cavity [12,13]. Because there were no clear causes of increase in IAP in our case, a discrepancy in pressure between the thoracic and abdominal cavities may have contributed to the development of this hernia. Moreover, the biopsy of pleura for mesothelioma also may result in the creation of a hernia orifice. Therefore, we diagnosed the patient with secondary (or iatrogenic) parahiatal hernia in our case.

On the one hand, laparoscopic repair for paraesophageal hernia has become widely recognized [14,15]. On the other hand, the number of reported cases of parahiatal hernia treated by laparoscopic surgery has increased gradually [1,2,4]. The procedures of hernia repair include primary repair or the use of a mesh. Rodefeld *et al*. [2] reported a case in which it was possible to perform the continuity hernia repair laparoscopically. They added laparoscopic Nissen fundoplication to reduce the risk of gastric volvulus in case of hernia recurrence. Scheidler *et al*. [4] reported a case in which both laparoscopic closure of a parahiatal hernia and standard Nissen fundoplication were performed. In their case, the normal location of the esophagogastric junction was revealed in a barium contrast study. Moreover, a preoperative esophageal manometric study revealed normal esophageal body peristalsis and normal resting pressure and length of the lower esophageal sphincter. The use of additional fundoplication as a prophylactic measure during the treatment of parahiatal hernia remains controversial because of the low incidence rate of this condition.

## Conclusions

We experienced an extremely rare case of parahiatal hernia that developed after treatment of a malignant mesothelioma and was successfully treated by laparoscopic surgery. Although the preoperative diagnosis of parahiatal hernia is difficult, a laparoscopic approach can be a useful therapeutic procedure, not only for paraesophageal hernia but also for parahiatal hernia.

## Consent

Written informed consent was obtained from the patient for publication of this case report and accompanying images. A copy of the written consent is available for review by the Editor-in-Chief of this journal.

## Competing interests

The authors declare that they have no competing interests.

## Authors’ contribution

All authors were actively involved in direct patient care and have read and approved the manuscript. MT is the principal author and was involved in the collection of data. KM contributed to writing the manuscript. TI and GH were involved in the collection of relevant literature and proof read the manuscript.

## References

[B1] Palanivelu C Rangarajan M Jategaonkar PA Parthasarathi R Balu K Laparoscopic repair of parahiatal hernias with mesh: a retrospective study Hernia 2008 12 521 525 10.1007/s10029-008-0380-2 18661099

[B2] Rodefeld MD Soper NJ Parahiatal hernia with volvulus and incarceration: laparoscopic repair of a rare defect J Gastrointest Surg 1998 2 193 197 10.1016/S1091-255X(98)80012-7 9834416

[B3] Trus TL Bax T Richardson WS Branum GD Mauren SJ Swanstrom LL Hunter JG Complications of laparoscopic paraesophageal hernia repair J Gastrointest Surg 1997 1 221 227 10.1016/S1091-255X(97)80113-8 9834351

[B4] Scheidler MG Keenan RJ Maley RH Wiechmann RJ Fowler D Landreneau RJ “True” parahiatal hernia: a rare entity radiologic presentation and clinical management Ann Thorac Surg 2002 73 416 419 10.1016/S0003-4975(01)03373-2 11845852

[B5] Demmy TL Boley TM Curtis JJ Strangulated parahiatal hernia: not just another paraesophageal hernia Ann Thorac Surg 1994 58 226 227 10.1016/0003-4975(94)91106-1 8037530

[B6] Vallieres E Waters PF Incarcerated parahiatal hernia with gastric necrosis Ann Thorac Surg 1987 44 82 83 10.1016/S0003-4975(10)62365-X 3606266

[B7] Flynn R Upside-down stomach in a parahiatal hernia Med J Aust 1953 1 925 926 13070935 10.5694/j.1326-5377.1953.tb85042.x

[B8] Ellis FH Jr Diaphragmatic hiatal hernias. Recognizing and treating the major types Postgrad Med 1990 88 113 114 1973284 10.1080/00325481.1990.11716365

[B9] Macdougall JT Abbott AC Goodhand TK Herniation through congenital diaphragmatic defects in adults Can J Surg 1963 6 301 315

[B10] Choi YU North JH Jr Diaphragmatic hernia after Ivor-Lewis esophagectomy manifested as lower gastrointestinal bleeding Am Surg 2001 67 30 32 11206892

[B11] Papavramidis TS Kotidis E Ioannidis K Cheva A Lazou T Koliakos G Karkavelas G Papavramidis ST The effects of chronically increased intra-abdominal pressure on the rabbit diaphragm Obes Surg 2012 22 487 492 10.1007/s11695-012-0587-2 22246392

[B12] Papavramidis TS Michalopoulos NA Mistriotis G Pliakos IG Kesisoglou II Papavramidis ST Abdominal compliance, linearity between abdominal pressure and ascitic fluid volume J Emerg Trauma Shock 2011 4 194 197 10.4103/0974-2700.82205 21769205 PMC3132358

[B13] Apostolidis S Papavramidis TS Michalopoulos A Papadopoulos VN Paramythiotis D Harlaftis N Groin swelling, the anatomic way out of abdominal haematomas: a case report and explicative literature review Acta Chir Belg 2008 108 251 253 18557154 10.1080/00015458.2008.11680214

[B14] Andujar JJ Papasavas PK Birdas T Robke J Raftopoulos Y Gagné DJ Caushaj PF Landreneau RJ Keenan RJ Laparoscopic repair of large paraesophageal hernia is associated with a low incidence of recurrence and reoperation Surg Endosc 2004 18 444 447 10.1007/s00464-003-8823-4 14752653

[B15] Geha AS Massad MG Snow NJ Baue AE A 32-year experience in 100 patients with giant paraesophageal hernia: the case for abdominal approach and selective antireflux repair Surgery 2000 128 623 630 10.1067/msy.2000.108425 11015096

